# Evaluation of tongue volume and oral cavity capacity using cone-beam computed tomography

**DOI:** 10.1007/s10266-017-0335-0

**Published:** 2018-02-21

**Authors:** Xuefang Ding, Shoichi Suzuki, Momotoshi Shiga, Naoto Ohbayashi, Toru Kurabayashi, Keiji Moriyama

**Affiliations:** 1 Department of Stomatology, Beijing Jishuitan Hospital, Beijing, China; 2 Maxillofacial Orthognathics, Graduate School, Tokyo Medical and Dental University, Tokyo, Japan; 3 Division of Orofacial Functions and Orthodontics, Kyushu Dental University, Kitakyushu-shi, Fukuoka-ken Japan; 4 Oral and Maxillofacial Radiology, Graduate School, Tokyo Medical and Dental University, Tokyo, Japan

**Keywords:** Tongue volume, Oral cavity capacity, Cone-beam CT, Radiocontrast agent, BMI

## Abstract

The aims of this study were to reveal the usefulness of a newly developed method for measuring tongue volume (TV) and oral cavity capacity (OCC) and to assess the relationship between them. The tongue was coated with a contrast agent, and the TV and OCC were determined using cone-beam computed tomography (CBCT). We enrolled 20 adults who were scheduled to undergo CBCT to evaluate the relationship of the third molar roots to the alveolar nerve before molar extraction. Each participant’s tongue was coated with a contrast agent, and CBCT of the tongue and oral cavity was performed. Using computer software, we evaluated reconstructed 3D images of the TV, oral cavity proper volume (OCPV), and OCC. The mean TV was 47.07 ± 7.08 cm^3^. The mean OCPV and OCC were 4.40 ± 2.78 cm^3^ and 51.47 ± 6.46 cm^3^, respectively. There was a significant correlation between TV and OCC (*r* = 0.920; *p* < 0.01) but not between TV and OCPV. The mean TV/OCC ratio was 91 ± 5%. The proposed method produced CBCT images that enabled effective measurement of TV and OCC. This simple method of measuring TV and OCC will be useful in the diagnosis on the tongues with abnormal size.

## Introduction

Teeth are aligned with the outer border of the tongue in a parabolic curve and are surrounded by cheek and lip muscles. Tooth position and the form of the dental arch are subject to constant pressure from the circumoral muscles and the tongue, and stable maintenance of the position of the teeth and the form of the dental arch is thought to depend on the balance of these pressures [1]. According to the equilibrium theory, the tongue in resting posture exerts a light force over a long duration, significantly influencing tooth position and the dental arch form [2, 3]. The volumetric relationship between the tongue and the oral cavity may be an important factor in tooth alignment and occlusion. Indeed, some types of malocclusion are known to be caused by volumetric discordance. For example, patients with macroglossia as a symptom of Beckwith–Wiedemann syndrome or acromegaly often have an anterior or lateral open bite [4, 5]. To preserve normal occlusion and tooth alignment, it is necessary to secure a certain proportion between the tongue volume (TV) and the oral cavity capacity (OCC).

Previous studies evaluating TV and OCC have used lateral cephalograms [6–8], alginate impressions [9, 10], computed tomography (CT), and magnetic resonance imaging (MRI) [11, 12]. Lateral cephalograms, however, do not show complex three-dimensional (3D) variations in the shape of the tongue and oral cavity. Although studies using CT and MRI have provided a better understanding of the 3D morphology of the tongue and oral cavity, the shape and position of the tongue are significantly influenced by gravity because the images are obtained while the patients are supine [13, 14]. The mobility of the tongue and the long exposure times needed for MRI may also result in motion artifacts, which decrease image quality.

Cone-beam computed tomography (CBCT) is useful for investigating the morphologic structures in the oral cavity because of its relatively low cost and availability in dental clinics. Its 3D image quality is comparable to that obtained with CT, but with a much lower effective radiation dose [15–17]. CBCT is advantageous because the patient is sitting upright, which prevents the tongue from falling backward under the influence of gravity. Additionally, the shorter exposure time results in fewer motion artifacts than MRI [18]. In 2011, Uysal et al. used stored CBCT data to measure TV and discussed the relationship between TV and lower incisor irregularity [19]. Because they measured the TV superior to the level of the cement–enamel junction of the lower teeth, the TV was dependent on the relative positions of the tongue and teeth. Moreover, it was difficult to visualize the border between soft tissue structures, such as between the lateral surface of the tongue and the lingual mucosa of the lower dental arch. Lauder et al. reported difficulty defining the inferior and lateral borders of the tongue on MRI, which is usually used for soft tissue imaging [20]. It is also difficult to visualize soft tissue borders on CBCT images because of the lack of contrast between the various soft tissue structures [21].

In the present study, the tongue and surrounding tissues were coated with a radiocontrast agent to enable clear identification of the soft tissue borders on the CBCT images. Subjects were also instructed to position the tongue in a uniform way. We measured TV and OCC and calculated the TV/OCC ratio using our proposed CBCT method. We also aimed to confirm the correlations between TV and OCC and between TV and BMI using the measurements acquired with this method. The purpose of this study was to reveal the usefulness of this new method for measuring TV and OCC using CBCT.

## Materials and methods

### Subjects

Participants were selected from patients scheduled to undergo CBCT to confirm the 3D relationship of their third molar roots and the inferior alveolar nerve canal after plain panoramic radiography suggested close proximity. It is difficult to include the area from the tooth roots of both sides of mandibular impacted third molars and whole tongue (from the lingual tip to the lingual root) in one take of cone-beam computed tomography (CBCT) imaging. Therefore, individuals with an impacted third molar in whom it was necessary to investigate the positional relationship between third molar root and inferior alveolar neural canal on one side only were selected as subjects.

A total of 20 adults (10 men, 10 women) with a mean age of 30.1 ± 2.3 years (range 26.5–34.7 years) were enrolled in the study. Participants were all Japanese. They had normal occlusion and had 1–3 mm anterior overjet and overbite. Their molar relationship was Angle Class I or nearly Class I. And all participants possessed the following criteria: no craniofacial deformity, no abnormal oral function, no missing teeth except for third molars, no severe crowding in the anterior teeth, and no history of maxillofacial surgery. The height and weight of each participant were measured at the time of CBCT, and their body mass index (BMI) was calculated. The Ethics Committee of Tokyo Medical and Dental University approved the study protocol (No. 885). All participants were informed of the purpose of the study and gave formal consent before undergoing CBCT.

### CBCT scans

CBCT was performed using a Fine Cube XP62 system (Yoshida Dental Manufacturing, Tokyo, Japan) at 90 kV, 4 mA, and a scan time of 8.6 s. The field of view was 81 × 74 mm^2^, and the isotropic resolution was 0.2 mm. Each participant’s head was positioned with the Frankfort plane parallel to the floor. The participant’s mouth was rinsed with a suspension of barium sulfate (BarytgenSol; Fushimi Pharmaceutical, Tokushima, Japan) to enable clear identification of the soft tissue borders of the tongue and surrounding tissues on the CBCT image. The surfaces of the tongue, especially the ventral and lateral surfaces, were coated with a stickier solution of barium sulfate to enhance the image. This solution was prepared by centrifuging the barium sulfate suspension twice at 4000×*g* for 10 min each time. The concentrated contrast agent was applied to the surface of oral bottom and around the tongue by using a brush. Each participant was instructed to relax and rest the tip of their tongue on the lingual surface of the lower incisors, with the mandible in the intercuspal position. CBCT was then performed.

### Segmentation and measurement

**Fig. 1:**
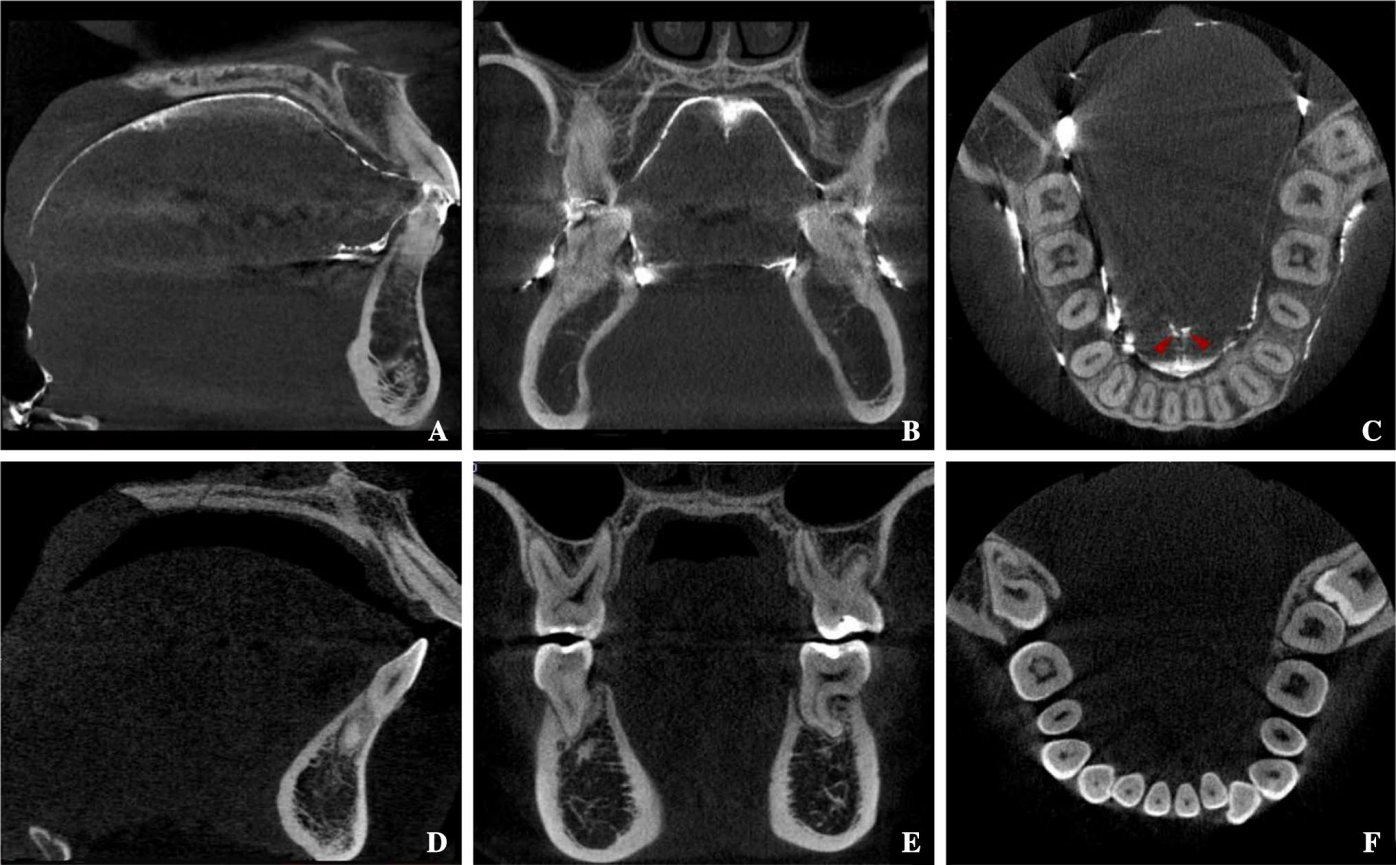
CBCT images of the oral cavity. **a**–**c** CBCT images of the oral cavity coated with radiocontrast agent. A clear border line is visible. Red arrows indicate the lingual frenulum. **d**–**f** CBCT images of the oral cavity without radiocontrast coating. The boundary of the tongue is indistinct, and the lingual frenulum is not distinguishable

**Fig. 2:**
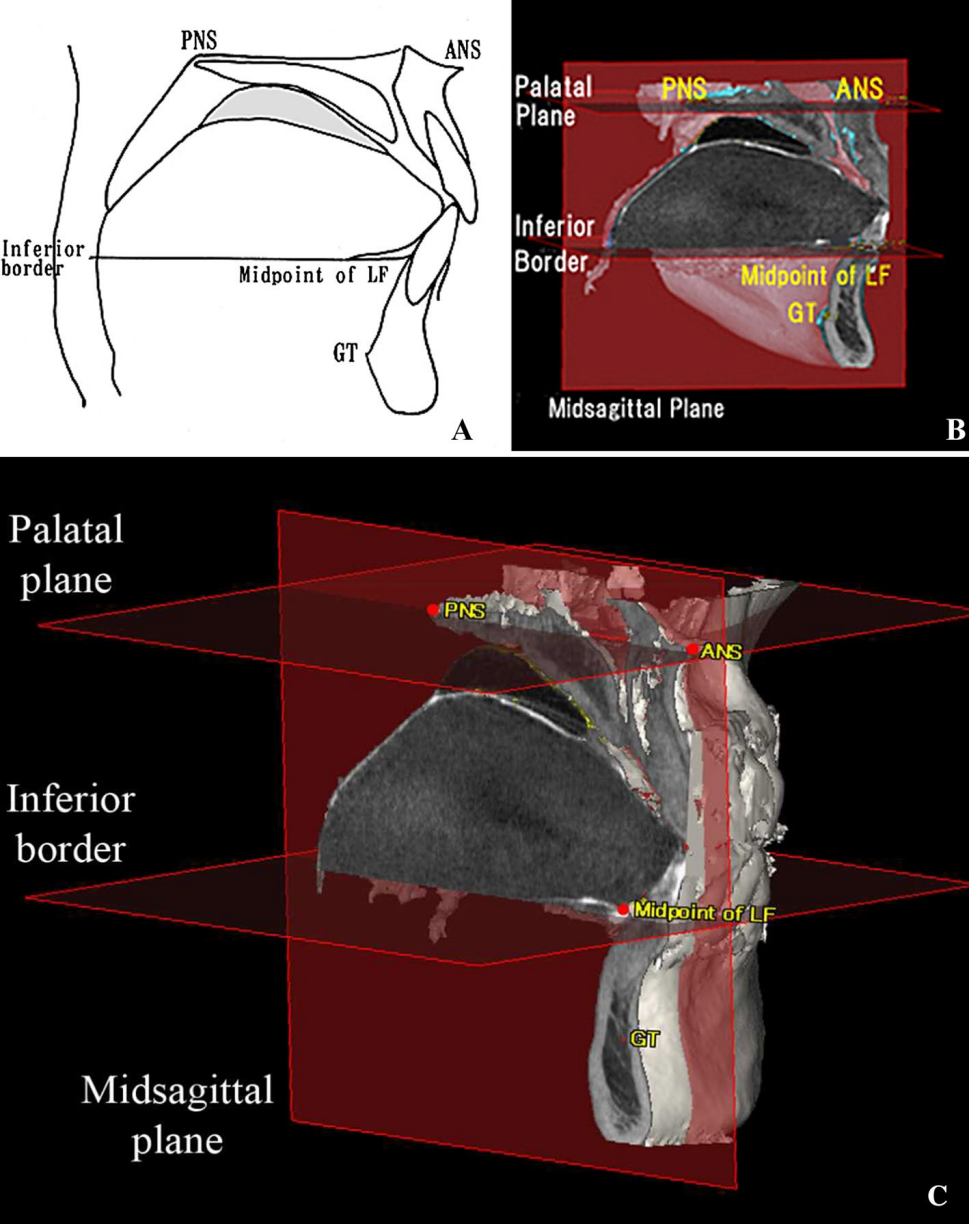
Landmarks, reference planes, and outlines for volume analysis. **a**, **b** Outlines in the mid-sagittal section, showing the area measured for the oral cavity proper and the tongue. **c** 3D view of the mid-sagittal section

**Fig. 3:**
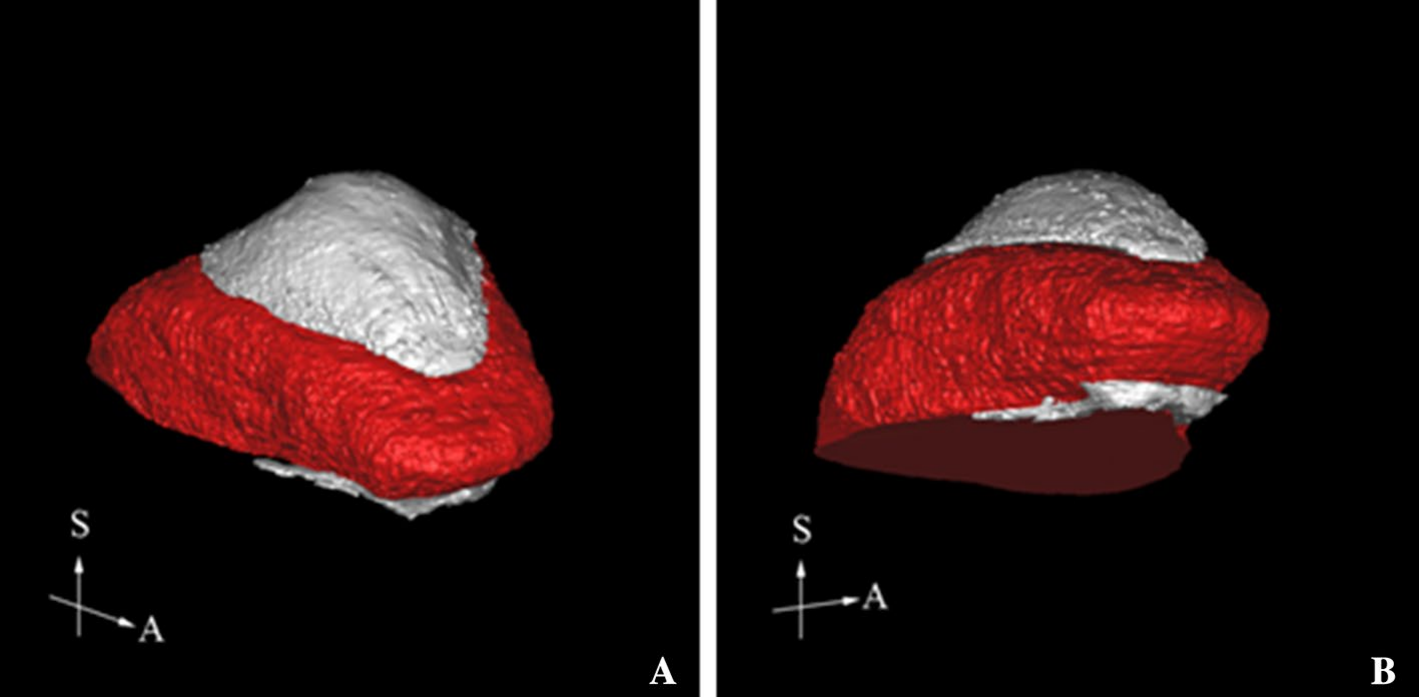
3D views of the tongue (red) and oral cavity proper (white)

Each participant’s Digital Imaging and Communications in Medicine (DICOM) data were imported into 3D image analysis software (SimPlant Crystal; Materialise Dental, Leuven, Belgium), providing 512 image slices of 0.147 mm thickness (Fig. 1). For volume analysis, the mid-sagittal plane was defined as the plane passing through the anterior nasal spine (ANS), posterior nasal spine (PNS), and the center of the genial tubercles. The palatal plane was defined as the plane passing through the ANS and PNS, perpendicular to the mid-sagittal plane. TV was measured superior to the attachment point of the lingual frenulum. The inferior border of the tongue was defined as the plane passing through the midpoint of the anterior margin of the lingual frenulum, parallel to the palatal plane. The posterior border of the tongue was determined by the radiopaque outlines seen in the images of posterior surface of tongue in the area of oropharynx. Tongue volume (TV) was measured as the part of tongue superior to the inferior border plane including the part of tongue in the oropharynx. On the other hand, oral capacity proper volume (OCPV) was included the space between the dorsum of tongue and palate and also included the space between the inferior surface of tongue and the floor of the mouth (Fig. 2). The outlines of the tongue and the oral cavity proper were traced manually on each slice. After reconstructing the 3D images, the TV and oral cavity proper volume (OCPV) were measured using 3D image analysis software (Fig. 3). The oral cavity was defined as the region that included the oral cavity proper and the tongue volume. The OCC was calculated as TV + OCPV, after which the TV/OCC ratio was calculated.

### Method error

Two phantoms (a cube and a cylinder), made of acrylic glass as a soft tissue equivalent, were used to test the accuracy of the volume measurements and to validate our method. The dimensions of the phantoms were measured to the nearest 0.1 mm using calipers. CBCT images of the phantoms were measured five times by the same observer using the method described above. To evaluate intraobserver variations in measurements, five participants were randomly selected, and the same observer repeated the measurements on these participants 2 weeks after the first measurements.

### Statistical analysis

The variables were found to have a normal distribution. A one-sample *t* test was used to evaluate the difference between the errors in the phantom measurements and zero. A paired-sample t test was used to analyze the differences between the repeated measurements in five participants. An independent samples t test was used to compare age and BMI between the male and female participants. We planned to control for sex-related differences in these variables. If there were no differences in age and BMI, the differences in TV, OCPV, OCC, and TV/OCC ratio for both sexes would be assessed using the independent samples *t* test. Pearson’s correlation test was used to analyze correlations among BMI, TV, OCPV, and OCC. A value of *p* < 0.05 was considered to indicate statistical significance. All analyses were performed using SPSS statistical software (version 13.0; SPSS Inc., Chicago, IL, USA).

## Results

### Method error

For the phantoms, the mean error in the volumetric measurements was 1.1 ± 2.1% (range − 4.5 to 2.0%).

### CBCT images of the tongue and surrounding structures

A CBCT image of the tongue is shown in Fig. 1. In the CT image of the oral cavity, the surface of the soft tissues, including the outline of the tongue, could barely be observed without the radiocontrasting coating (Fig. 1d–f). In particular, the borders of the adjacent soft tissues (such as between the lateral border of the tongue and the lingual gingiva of the lower molars, and between the inferior border of the tongue and the lingual gingiva of the lower incisors) were barely distinguishable in the CBCT image without the barium sulfate coating (Fig. 1e, f). However, 3D morphology of the tongue was clearly discernable in the CBCT image with the barium sulfate coating (Fig. 1b). Additionally, the position of the tongue frenulum was clearly confirmed by visualizing the coated inferior surface of the tongue and the base of the oral cavity (Fig. 1a, c).

### Measurement of TV, OCPV, and OCC

For the phantoms, the mean error between the actual volume and the estimated volume determined from the CBCT images was − 1.1 ± 2.1% (*p* = 0.126), indicating that the processes of scanning, segmentation, and measurement were accurate.

For the five cases that were re-measured after 2 weeks, the first and second measurements were not significantly different (TV: *p* = 0.130; OCPV: *p* = 0.681), indicating excellent intraobserver reliability (Table 1). Comparisons of age, BMI, TV, OCPV, OCC, and TV/OCC ratio between males and females are shown in Table 2. None of these characteristics were significantly different between males and females. There were significant correlations between BMI and TV (*r* = 0.502; *p* = 0.024) and between BMI and OCC (*r* = 0.547; *p* = 0.013), but there was no significant correlation between BMI and TV/OCC ratio (*p* = 0.757). There was a significant correlation between TV and OCC (*r* = 0.920; *p* < 0.01), but there was no significant correlation between TV and OCPV (*p* = 0.072) (Table 3).

**Table 1:** Comparisons and correlations of repeated measurements

	Differences	*t*	*p*	*r*	*p*
	Mean ± SD				
TV (cm^3^)	− 1.11 ± 1.30	− 1.904	0.130	0.959	0.010*
OCPV (cm^3^)	0.03 ± 0.13	0.442	0.681	0.999	0.000**

*TV* tongue volume; *OCPV* oral cavity proper volume

*p < 0.05; **p < 0.001

**Table 2:** Comparisons of characteristics and measurements between males and females

	Mean ± SD	Male	Female	*F*	*p*	*t*	*p*
Age (year)	30.1 ± 2.3	30.8 ± 2.2	29.4 ± 2.3	0.355	0.559	1.315	0.205
BMI (kg/m^2^)	21.4 ± 2.6	21.9 ± 2.9	20.9 ± 2.4	1.308	0.268	− 0.836	0.414
TV (cm^3^)	47.07 ± 7.08	49.18 ± 7.72	44.97 ± 6.04	0.672	0.423	1.356	0.192
OCPV (cm^3^)	4.40 ± 2.78	3.56 ± 2.37	5.23 ± 3.03	0.028	0.869	− 1.366	0.189
OCC (cm^3^)	51.47 ± 6.46	52.74 ± 6.79	50.20 ± 6.20	0.004	0.950	0.874	0.394
*R* (%)	91.4 ± 5.4	93.1 ± 4.7	89.6 ± 5.7	0.064	0.804	1.455	0.163

*TV* tongue volume; *OCPV* oral cavity proper volume; *OCC* oral cavity capacity; *R* ratio of tongue volume to oral cavity capacity; *BMI* body mass index

**Table 3:** Correlations between TV and BMI, OCC and BMI, *R* and BMI, TV and OCPV, and TV and OCC

	TV versus BMI	OCC versus BMI	*R* versus BMI	TV versus OCPV	TV versus OCC
*r*	0.502	0.547	0.074	− 0.411	0.920
*p*	0.024*	0.013*	0.757	0.072	0.000**

*TV* tongue volume; *OCPV* oral cavity proper volume; *OCC* oral cavity capacity; *R* ratio of tongue volume to oral cavity capacity; *BMI* body mass index

*p < 0.05; **p < 0.001

## Discussion

Lauder et al. compared rabbit TV estimated from MRI images with the actual TV and reported an error of − 4.3 ± 13.2% on coronal sections and − 5.9 ± 8.4% on sagittal sections [20]. Aboudara et al. measured airway phantoms on CBCT images and reported a mean error of 5.00 ± 0.23% for the angled airway phantom [22], which is comparable to a mean error of 1.1 ± 2.1% (range − 4.5 to 2.0%) for the volumetric measurements of the phantoms in our study. The volumetric measurements on CBCT images with contrast agent are therefore considered to be accurate and reliable.

As the tongue position may influence TV measurement, a normal, stable tongue position should be used for CBCT. Previous studies reported that the normal tongue position with the mandible in the intercuspal position was reproducible in individuals [23–25]. The normal position was usually with the tip of the tongue in contact with the lingual surfaces of the lower incisors, although positioning with the tip in contact with both upper and lower incisors was also observed [23, 24, 26]. To obtain a standardized tongue position in this study, subjects were instructed to position the tip of the tongue so it contacted the lingual surfaces of the lower incisors.

In this study, the mean TV was 47.07 ± 7.08 cm^3^, which is larger than the TV reported by Uysal et al. (31.02 ± 9.75 cm^3^ for men and 28.13 ± 8.54 cm^3^ for women). Uysal et al. defined the lower border of the tongue as the plane of the cement–enamel junction of the lower first molars and premolars, and the posterior borders as the plane descending from the PNS on the axial view [19]. Because the area measured was the upper part of the tongue superior to the plane of the cement–enamel junction of the lower molars, the TV values measured were smaller than those measured in our study. If TV were defined as the upper part of the tongue volume superior to the plane of the cement–enamel junction of the lower molars, the TV measurements might change according to the relative positional relationship between the tongue and lower molars. In the present study, TV was measured as the tongue volume superior to the plane passing through the midpoint of the anterior margin of the lingual frenulum, parallel to the palatal plane. According to this definition, it was thought that the TV measurement would not change with different relative positional relationships between the tongue and the lower molars. Humbert et al. reported a TV of 63.3 ± 8.2 cm^3^ measured on MRI, including all the intrinsic and extrinsic muscles of the tongue (e.g., hyoglossus and styloglossus) [27]. Our TV measurements are smaller than those of Humbert et al. because we measured the area of the tongue superior to the level of the attachment point of the lingual frenulum. As the tongue is composed of soft tissue with flexibility in its shape and position, it might be difficult to establish a particular region of interest in the tongue according to its intrinsic anatomic structure. Based on our 3D coordinate system constructed in the CBCT image, TV was defined as the area of the tongue surrounded by the palate and the upper and lower dental arches in this study. This area is recognized as “tongue” intuitively and most commonly, and it is thought to have the greatest effect on the form of the dental arch, including its width and length, and also to contribute to occlusion. Thus, our results are consistent with those of Uysal et al. but are different from those of others [12, 19, 20, 27–32].

A correlation between TV and BMI has been reported in both healthy adults and patients with obstructive sleep apnea syndrome [30, 31, 33], and a similar correlation was found in this study. Nashi, in an autopsy study, reported that tongue weight was correlated with BMI [34]. The tongue has a much higher percentage of fat than other somatic muscles, and its fat content increases with increasing BMI. The correlation between TV and BMI in the present study may reflect a correlation between tongue fat content and BMI. We also found a correlation between TV and OCC. The mean *R* value was 91.4 ± 5.4%. Lauder et al. estimated the volume of the tongue and oral cavity using MRI, by defining: (1) the inferior border of the tongue as the line from the genial tubercle to the hyoid bone; (2) the posteroinferior border of the tongue as the line from the hyoid bone to the vallecula; and (3) the oral cavity as the area of the tongue plus the oral cavity proper and the oropharynx. They reported that the ratio of TV to the oral cavity was about 91% [20]. Iida-Kondo et al. reported a TV/OCC ratio of 86.98%, which is similar to ours, even though they used different definitions of the tongue and oral cavity for the measurements [12]. Consistent with our data, they also reported a positive correlation between TV and OCC, suggesting that the volume of the tongue and oral cavity are related to each other. For example, if the tongue is large, the oral cavity is also expected to be large. On clinical examination, individuals with a large tongue (e.g., those with acromegaly) almost always have a well-developed mandible [35]. However, there was no significant correlation between the OCPV and TV, suggesting that the oral cavity proper, the space around the tongue, and the position of the tongue are not strongly affected by TV, but can be considered to reflect the tongue position relative to the maxilla and mandible. In this study, the OCPV was 0.54–11.14 cm^3^, although some studies describing the “in-mouth air cavity” or “intraoral airway” (both areas similar to the oral cavity proper) varied from 0.42 ± 0.80 to 12.13 cm^3^ [36–39]. The large variation in OCPV may result from individual differences in habitual tongue positioning.

The method proposed in this study showed that TV and OCPV could be measured clearly on 3D CBCT images using a contrast agent. This method for measuring TV and OCC would be helpful for estimating the degree of tongue enlargement in patients with macroglossia as a symptom of Beckwith–Wiedemann syndrome or acromegaly. Should glossectomy be needed in these patients, TV and OCC measurements would supply the correct TV/OCC ratio for planning the extent of the tongue resection. CBCT is expected to be widely used for various imaging-based analyses in the oral region. The use of a radiocontrast agent allows CBCT to be used not only in skeletal analysis, but also in soft tissue analysis.
